# Supplementary material for: Mini-MEndR: a miniaturized 96-well predictive assay to evaluate muscle stem cell-mediated repair
Source: BMC Methods. 2024 Jun 7;1(1):5. doi: 10.1186/s44330-024-00005-4 (PMC11173370; doi:10.1186/s44330-024-00005-4)
Supplement: Supplementary file 1 — Supplementary Material 1. [file 44330_2024_5_MOESM1_ESM.docx]

**SUPPLEMENTARY INFORMATION**

**mini-MEndR: A miniaturized 96-well predictive assay to evaluate muscle stem cell-mediated repair**

Nitya Gulati^1,2†^, Sadegh Davoudi^2,3†^, Bin Xu^2,3†^, Saifedine T. Rjaibi^1-3‡^, Erik Jacques^2,3‡^, Justin Pham^1,2^, Amir Fard^1-3^, Alison P. McGuigan^1,3*^, Penney M. Gilbert^2-4*^

^1^ Department of Chemical Engineering and Applied Chemistry, University of Toronto, Toronto, ON, M5S3E4, Canada

^2^ Donnelly Centre, University of Toronto, Toronto, ON M5S3E1, Canada

^3^ Institute of Biomedical Engineering, University of Toronto, Toronto, ON, M5S3G9, Canada

^4^ Department of Cell and Systems Biology, University of Toronto, Toronto, ON M5S3G5, Canada

^*^ Co-corresponding author e-mails: [penney.gilbert@utoronto.ca](mailto:penney.gilbert@utoronto.ca) (P.M.G.) and [alison.mcguigan@utoronto.ca](mailto:alison.mcguigan@utoronto.ca) (A.P.M.)

^†^ These authors contributed equally to this work.

^‡^ These authors contributed equally to this work.

**Supplementary Figures 1-5**

**Supplementary Tables 1-5**

**Supplementary Figure 1. Pluronic acid coating allows for the generation of smooth mini-myotube template edges.** Representative confocal images of mini-myotubes generated with (+) and without (-) pluronic acid coating. Tissues are immunostained for sacromeric α-actinin (SAA; magenta). Scale bar, 1 mm.

**Supplementary Figure 2. Mini-MEndR platform manufacture can be quickly mastered by new users. (A)** Representative confocal images of myotube templates generated by the same user at different levels of experience. Tissues were fixed after 7 days of differentiation and stained with phalloidin (magenta). Scale bar, 500 μm. **(B)** Quantification of Phalloidin (User 1) or SAA (User 2, 3) tissue coverage attained by three different users who trained from first-time user to expert level as indicated by plateauing coverage and homogeneous mini-myotube templates. Coverage was normalized to the average of each user’s three most recent seeding attempts at expert level to account for cell line-to-cell line differences in absolute fiber coverage.

**Supplementary Figure 3. Gating strategy used for MACS or FACS based MuSCs isolation. (A)** Schematic showcasing the workflow of MACS vs FACS MuSC isolation protocols. **(B)** Flow cytometry plots demonstrating gating strategy to enrich MuSCs from Pax7-nGFP transgenic mice using FACS. **(C)** Representative plots of green fluorescent protein (GFP)^+^ cells present in MuSC preparations derived from Pax7-nGFP tissue samples using FACS (left) as compared to MACS (right)**. (D)** Enumeration of % total GFP^+^ cells enriched from Pax7-nGFP tissue samples using a variety of optimisations that include the number of lineage depletion column rounds with or without an integin-α7 column enrichment round performed during the MACS protocol.

**Supplementary Figure 4. MACS and FACS sorted MuSCs are functionally similar in mini-MEndR assays. (A)** Representative confocal images and **(B)** quantification of yellow fluorescent protein (YFP; yellow) immunostaining coverage of 18M mini-MEndR tissues at 10 DPI that were generated with FACS or MACS sorted MuSCs and treated with DMSO. Scale bar, 500 $\mu$m. Graphs display mean ± s.e.m.; unpaired two-tailed T-test with Welch's correction, ns = no significance. n=9-10 tissues from N = 3 independent experiments per cell line.

**Supplementary Figure 5. mini-MEndR assay demonstrates return to quiescence dynamics by a subset of donor-derived cells by day 7 post-injury.** Quantification of Pax7^+^ GFP^+^ EdU^-^ cells in mini-MEndR tissues generated with 18M (left) and 19F (right) cell lines, following treatment with DMSO or p38α/β MAPKi in the context of injury and analyzed at 7 DPI. Graphs display mean ± s.e.m.; Unpaired two-tailed T-test with Welch’s correction. ns p > 0.5, * p < 0.5, n=5-6 tissues and 3-5 images per tissue from N=2 independent experiments per cell line.

**Supplementary Table 1. Human skeletal muscle myoblast donor information**

| **Age** | **Sex** | **Ethnicity** | **Vendor** | **Catalog #** | **Tissue of Origin** | **Additional Information** |
| --- | --- | --- | --- | --- | --- | --- |
| 18 | M | Caucasian | Cook MyoSite | SK-1111-P01236-18M | Rectus Abdominus | Active |
| 18 | F | Caucasian | Cook MyoSite | SK-1111-P01431-18F | Vastus Lateralis | Healthy, no known medical conditions |
| 19 | F | Caucasian | Cook MyoSite | SK-1111-P01358-19F | Vastus Lateralis | N/A |

**Supplementary Table 2. Culture medium and solutions**

| **Media** | **Composition** |
| --- | --- |
| Wash Medium | 10 % FBS, 90 % DMEM |
| Human pMB Growth Medium | Ham’s F-10 nutrient mix, 20 % Foetal bovine serum FBS, 5 ng/mL rh-FGF2, 1 % Penicillin-Streptomycin |
| 2D Differentiation Media | DMEM, 2 % Horse serum, 10 mg/mL insulin (Sigma, #I6634), 1 % Penicillin-Streptomycin |
| Rat Tail Collagen Solution | 0.1 M Acetic Acid, Collagen I Rat Tail Stock solution (Gibco #A1048301), 0.1 M Acetic Acid, Collagen Stock Solution |
| MuSC Growth Medium | DMEM/F12, 1 % Pen-strep (P/S), 20 % Foetal bovine serum (FBS), 10 % Horse serum (HS), 1 % 2 mM Q (Glutamax), 1 % Insulin-Transferrin-Selenium (ITS), 1 % Non-Essential Amino acids, 1 % 1 mM Sodium Pyruvate, 50 uM (1/1,000) β-mercaptoethanol, 5 ng/ml (1/5,000) Gibco FGF2 |
| MEndR Growth Medium | Ham’s F-10 nutrient mix, 20 % FBS, 1.5 mg/mL 6-Aminocaproic Acid (ACA, Sigma, #A2504), 1 % Penicillin-Streptomycin |
| MEndR Differentiation Medium | DMEM, 2 % Horse serum, 2 mg/mL ACA, 10 mg/mL insulin (Sigma, #I6634), 1 % Penicillin-Streptomycin |
| Fibrinogen Solution | 10 mg/mL Fibrinogen (Sigma, #F8630) in NaCl solution (0.9 % wt/vol in ddH2O) |
| Extracellular Matrix (ECM) Master Mix | 40 % DMEM, 40 % Fibrinogen, 20 % Geltrex^TM^ |
| Red Blood Cell Lysis Buffer | H_2_O, 0.155 M NH_4_Cl, 0.01 M KHCO_3_, 0.1 mM EDTA |
| FACS Buffer | PBS, 2.5 % goat serum, 2 mM EDTA |
| Blocking Solution | PBS, 10 % goat serum, 0.3 % Triton X-100 (BioShop, #TRX777) |

**Supplementary Table 3. Antibodies and other reagents**

| **Antibody** | **Host species** | **Dilution** | **Manufacturer** |
| --- | --- | --- | --- |
| Alexafluor® 647 Anti-Human CD56 | Mouse | 1:20 (FC) | BD, #557711 |
| eFluor® 660 Anti-Mouse CD34 | Rat | 1:66 (FC) | eBioscience, #50034182 |
| PE Anti-Mouse α7-Integrin | Rat | 1:500 (FC) | AbLab, #530010-05 |
| Biotin Anti-Mouse CD31 | Rat | 1:200 (FC) | BD, #553371 |
| Biotin Anti-Mouse CD45 | Rat | 1:500 (FC) | BD, #553078 |
| Biotin Anti-Mouse CD11b | Rat | 1:200 (FC) | BD, #553309) |
| Biotin Anti-Mouse Ly-6A/E | Rat | 1:200 (FC) | BD, #553334 |
| Propidium Iodide | - | 1:1000 | Sigma, # P4864 |
| Anti-Sarcomeric α-actinin | Mouse | 1:500 | Sigma, #A7811 |
| Anti-GFP | Rabbit | 1:500 (IF) | ThermoFisher, #A11122 |
| Anti-Pax7 | Mouse | 1.5:1 (IF) | DSHB Pax7 hybridoma |
| Alexafluor® 488 Anti-Rabbit | Goat | 1:500 (IF) | ThermoFisher, #A11008 |
| Alexafluor® 546 Anti-Mouse | Goat | 1:500 (IF) | ThermoFisher, #A11003 |
| Alexafluor® 647 Anti-Rat | Goat | 1:500 (IF) | ThermoFisher, #A21247 |
| Phalloidin 568 | - | 1:500 (IF) | Life Technologies, #A12380 |
| Hoechst | - | 1:1000 (IF) | ThermoFisher, #H3570 |
| DRAQ5 | - | 1:1000 (IF) | Cell Signaling Technology, #4084L |
| DAPI | - | 1:1000 (IF) | Roche, #10236276001 |
| Satellite Cell Isolation Kit | Mouse | 1:5 (MACS) | Miltenyi Biotec, #130-104-268 |
| Anti-Integrin-α7 Beads | Mouse | 1:5 (MACS) | Miltenyi Biotec, #130-104-261 |
| Click-iT^TM^ Plus EdU Cell Proliferation Kit |  | (EdU, 1 μM) | ThermoFisher, #C10638 |

**Supplementary Table 4. Small molecule information**

| **Drug name** | **Target** | **Working concentration** | **Manufacturer** |
| --- | --- | --- | --- |
| SB203580 | p38α/β MAPK | 10 μM | New England Biolabs, #5633 |
| DMSO | - | - | Sigma Aldrich, #D2650 |

**Supplementary Table 5. Statistical tests and analysis**

| Figure | Biological experiments (N) | Total tissues/wells per condition (n) | Total images/wells per tissue | Statistical tests |
| --- | --- | --- | --- | --- |
| 1C | 1 per cell line | 3 | 5 | Ordinary one-way ANOVA with Tukey’s multiple comparison’s test |
| 1D | 1 per cell line | 3 | 5 | Ordinary one-way ANOVA with Tukey’s multiple comparison’s test |
| 1E | 1 per cell line | 3 | 5 | Ordinary one-way ANOVA with Tukey’s multiple comparison’s test |
| 1H | 1 per cell line | 2 | 3 | Ordinary one-way ANOVA with Tukey’s multiple comparison’s test |
| 2C | 2 | 8 | 1 | Two-way ANOVA with Sidak’s multiple comparisons test |
| 2F | Mixed in: 3  Pre-adsorbed: 4 | Mixed in: 11  Pre-adsorbed:24 | 1 | Unpaired two-tailed t-test |
| 3C | 3 | Day 7: 9  Day 10: 10  Day 14: 10 | 1 | Ordinary one-way ANOVA with Tukey’s multiple comparison’s test |
| 3D | 3 | Day 7: 9  Day 10: 8  Day 14: 6 | 1 | Ordinary one-way ANOVA with Tukey’s multiple comparison’s test |
| 4C | 6 | CTX (-): 18  CTX (+): 20 | 1 | Unpaired t-test |
| 4D | 5 | CTX (-): 15  CTX (+): 16 | 1 | Unpaired t-test |
| 5C | 5 | DMSO CTX (-):15  DMSO CTX (+):15  p38i CTX (+):15 | 1 | Ordinary one-way ANOVA with Tukey’s multiple comparison’s test |
| 5D | 4 | DMSO CTX (-):12  DMSO CTX (+):12  p38i CTX (+):12 | 1 | Ordinary one-way ANOVA with Tukey’s multiple comparison’s test |
| 6C | 4 | DPI7 DMSO: 11  DPI7 p38i:12  DPI10 DMSO:11  DPI10 p38i:10 | 1 | Multiple t-tests with Welch’s correction |
| 6D | 3 | DPI7 DMSO: 9  DPI7 p38i:9  DPI10 DMSO:8  DPI10 p38i:9 | 1 | Multiple t-tests with Welch’s correction |
| 7C | 3 | DMSO: 6  p38i: 8 | 4-7 | Unpaired two-tailed T-test with Welch’s correction |
| 7D | 3 | DMSO: 7  P38i: 8 | 4-7 | Unpaired two-tailed T-test with Welch’s correction |
| 7E | 3 | DMSO: 6  p38i: 8 | 4-7 | Unpaired two-tailed T-test with Welch’s correction |
| 7F | 3 | DMSO: 7  P38i: 8 | 4-7 | Unpaired two-tailed T-test |
| SI 4B | 3 | FACS: 9  MACS: 10 | 1 | Unpaired two-tailed T-test with Welch's correction |
| SI 5A | 2 | DMSO: 5  p38i: 6  for both 18M and 19F | 3-5 | Unpaired two-tailed T-test with Welch's correction |
